# Survival rate of colorectal cancer in China: A systematic review and meta-analysis

**DOI:** 10.3389/fonc.2023.1033154

**Published:** 2023-03-03

**Authors:** Ren Wang, Jie Lian, Xin Wang, Xiangyi Pang, Benjie Xu, Shuli Tang, Jiayue Shao, Haibo Lu

**Affiliations:** Department of Outpatient Chemotherapy, Harbin Medical University Cancer Hospital, Harbin, China

**Keywords:** colorectal cancer, overall survival, meta-analysis, China, epidemiology

## Abstract

**Background:**

This study aims to comprehensively summarize the colorectal survival rate in China. Method: In PubMed and Web of Science, keywords such as “colorectal cancer”, “survival” and “China” were used to search literatures in the past 10 years. Random effect models were selected to summarize 1-year, 3-year, and 5-year survival rates, and meta-regression and subgroup analyses were performed on the included studies.

**Results:**

A total of 16 retrospective and prospective studies providing survival rates for colorectal cancer in China were included. The 1-year, 3-year, and 5-year survival rates of colorectal cancer in China were 0.79, 0.72 and 0.62, respectively. In the included studies, the 5-year survival rates of stage I (5474 cases), stage II (9215 cases), stage III (8048 cases), and stage IV (4199 cases) colorectal cancer patients were 0.85, 0.81, 0.57 and 0.30, respectively. Among them, the 5-year survival rates of colorectal cancer were 0.82, 0.76, 0.71, 0.67, 0.66, 0.65 and 0.63 in Tianjin, Beijing, Guangdong, Shandong, Liaoning, Zhejiang and Shanghai, respectively.

**Conclusion:**

The 5-year survival rate in China is close to that of most European countries, but still lower than Japan and South Korea, and the gap is gradually narrowing. Region, stage, differentiation, pathological type, and surgical approach can affect 5-year survival in colorectal cancer.

**Systematic review registration:**

https://www.crd.york.ac.uk/prospero/ identifier, CRD42022357789.

## 1 Introduction

Colorectal cancer (CRC) is the third most common cancer in the world. In 2020, there were 1.9 million new cases of CRC and 93,5000 related deaths (1) Currently, China is undergoing cancer transition with an increasing burden of gastrointestinal cancer. The incidences of CRC increased rapidly (2). In recent years, the economic burden associated with CRC has been rising. Studies have shown that CRC-related healthcare spending is growing rapidly, with overall direct healthcare expenditure per CRC patient in China exceeding GDP per capita in the same year (3). The proportion of rectal cancer in China decreased from 71.2% in the 1980s to 66.7% in the 1990s, while the proportion of colon cancer increased from 10.9% to 15.2% during the same period. In China, the incidence of left and right CRC is basically the same. Among all CRC patients, 49.2% are observed on the right side and 49.4% are observed on the left side. Adenocarcinoma remains the most common type of CRC (4).CRC-related genetic syndromes, such as Lynch syndrome and familial adenomatous polyposis are responsible for 5%−10% of all CRC cases (5). A retrospective study from China showed that Lynch syndrome is an autosomal dominant hereditary Disease, representing 4% of all CRC cases (6).

Survival rate is one of the most critical indicators to measure the therapeutic effect and prognosis of a certain disease. Many social factors will affect the survival of colorectal cancer patients, and the disease itself, such as stage, differentiation, pathological type, tumor site, inflammatory factor, age and gender, will also affect survival (7). The 5-year survival rate of patients with stage I colon cancer was as high as 96.6%, while the 5-year survival rate of patients with stage IV colon cancer was only 34.3% (8). Research has reported that younger patients have a worse prognosis than older patients (9). A single-institution retrospective study showed better survival after radical resection of left colon cancer than right colon cancer, with a significant difference in 5-year overall survival between right and left colon cancer (82.1% *vs*. 88.7%, *P* < 0.05) (10). In the past few decades, the survival rate of CRC patients has improved significantly with the improvement of diagnostic technology and treatment, but there are still significant regional differences in the survival rate of CRC patients across the country.

There are many researches on the survival rate of CRC in China, providing valuable experience for the treatment and prognosis by understanding the survival rate of colorectal cancer in different regions and at different times. The purpose of this study is to analyze and summarize the survival rate of CRC in China.

## 2 Materials and methods

### 2.1 Study design

This systematic review and meta-analysis followed the Preferred Reporting Items for Systematic Reviews and Meta-analyses (PRISMA). By applying the Problem/Population, Intervention, Comparison, and Outcome (PICO) framework, the patients involved in our meta-analysis were colorectal cancer patients. We do not have a specific definition of “Intervention”. Articles that provided survival were included. In all included studies, the “Comparison” element of the PICO framework was not involved because we performed a pooling of single-group rates. The primary outcomes were 1-year, 3-year, and 5-year survival rates.

### 2.2 Search strategy

This systematic review and meta-analysis followed the Preferred Reporting Items for Systematic Reviews and Meta-analysis (PRISMA) statements checklist. A comprehensive, computerized literature search was conducted in PubMed and Web of Science for relevant studies between January 2011 and December 2021. The keywords and MeSH terms we used for retrieval were: (colorectal) or (colon) or (rectum) and (Neoplasms) or (tumor) or (cancer) or (Malignancy) or (carcinoma) and (survival) or (survival rate) or (survival analysis) or (prognosis) and (China). The search strategy was repeatedly performed until no new relevant articles were found. In addition, we reviewed references in the retrieved articles to search for additional relevant studies. All articles were evaluated by two authors based on the eligibility criteria we designed.

### 2.3 Selection of researches

First, we checked titles and abstracts of articles that were searched by using keywords to exclude irrelevant articles. Then the retrieved literatures were imported into “EndNote X9” software to exclude the duplicated ones before being screened by two reviewers independently. The exclusion criteria for our studies were as follows. (1) Studies were published in the year before 2011. (2) Articles that do not accurately provide complete survival information. (3) Studies with a sample size of less than 500 patients. (4) Article type is meta-analysis or review. (5) The language of the article is non-English.

### 2.4 Quality evaluation and data extraction

We evaluated comparative studies using the Newcastle-Ottawa Quality Assessment Scale for Cohort Studies. For the methodological quality evaluation of randomized controlled trials, we took reference to the Cochrane Handbook for Systematic Reviews of Interventions. Data were extracted independently by two researchers and any discrepancies in the data were settled by consensus. If necessary, a third researcher was expected to participate in the discussion and make a decision.

Data were extracted independently by two researchers using pre-designed standard forms, including corresponding author, study design, publication year, research year, region of patients, number of patients, age, sex, tumor site, tumor stage, differentiation, pathological type, surgical approach and 1-, 3- and 5-year survival rates. All data were extracted directly from the original text or calculated from the data known in the original text.

### 2.5 Data analysis

Firstly, we generated combined 1-, 3- and 5-year survival rates. Second, we performed subgroup analysis and meta-regression analysis when heterogeneity existed between the studies. Subgroup analysis was performed based on the factors of study region, stage, differentiation, pathological type, surgical approach, age and gender. The 5-year survival rates of different subgroups were calculated. The factors of sample size, publication year, research year and study region were included into the meta-regression model for meta-regression analysis. Sensitivity analysis was performed to investigate the risk of publication bias for individual studies. Finally, Egger bias test and funnel plot were used to evaluate the risk of publication bias in the included researches. We used Stata13 for meta-analysis and Graphpad for mapping.

## 3 Results

### 3.1 Included researches and their characteristics

**Figure 1:**
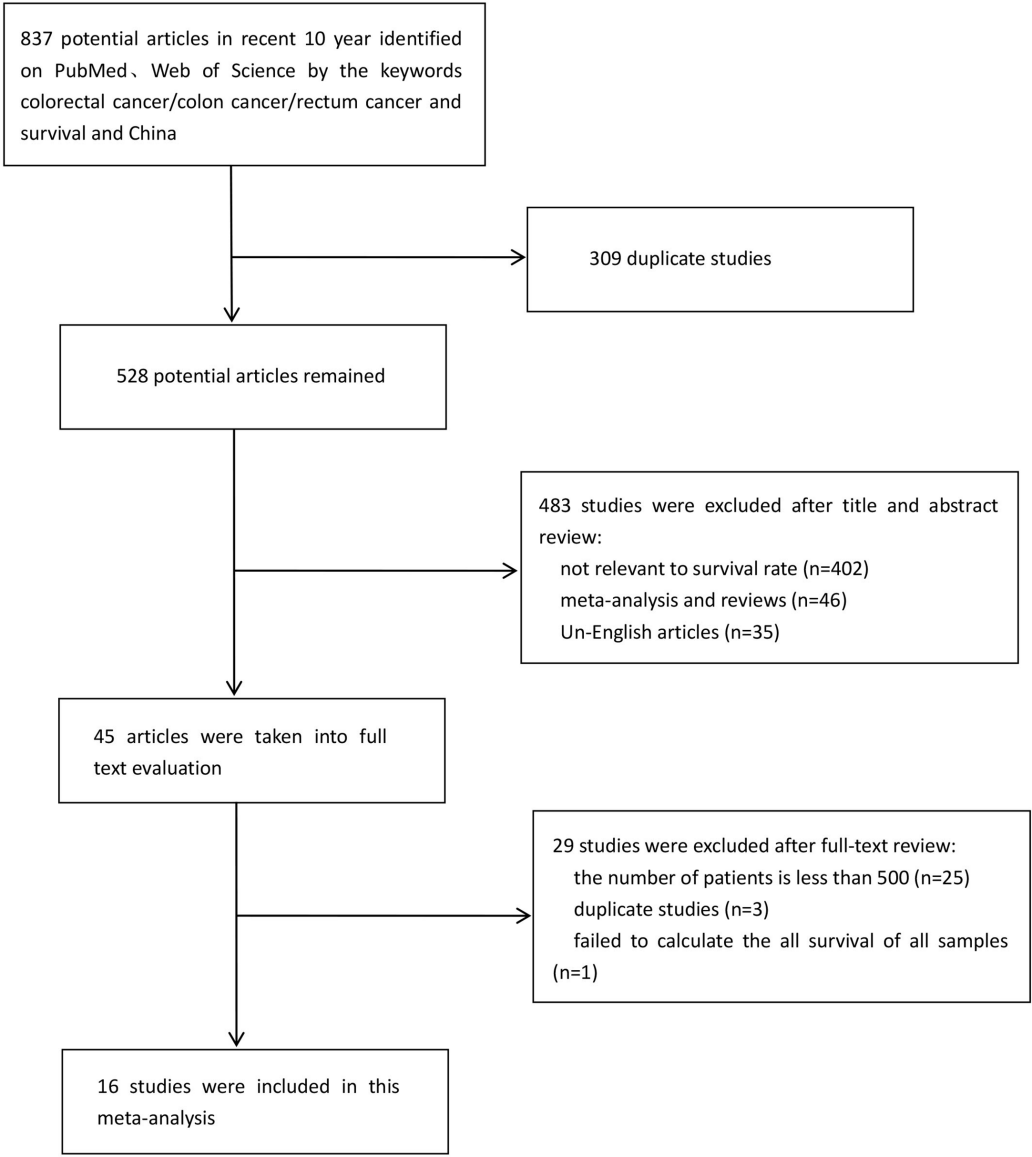
Flowchart of Search and Studies Selection.

Sixteen researches were eventually included in the meta-analysis (11–26). The combined search identified 837 literatures published, of which 309 duplicates were excluded. 483 were rejected based on the title and abstract evaluation (402 articles did not mention survival rate, 46 articles were meta-analyses and reviews, and 35 articles were non-English), and the remaining 45 articles underwent full-text evaluation. In order to minimize publication bias and make the included researches more representative, we excluded 25 researches with the number of cases fewer than 500. Three researches (13, 27, 28) were proven to be based on the same patient population, so only the one with the most comprehensive information was included (13). There were two researches (26, 29) with the same situation, and we excluded the one with less comprehensive information (29). One epidemiological research was excluded because it was unable to calculate overall survival rate of CRC patients. A flow diagram of the literature selection process used in this study was shown in **Figure 1**. The 16 researches with 62,748 patients, including 4 from Shanghai, 2 each from Zhejiang and Shandong provinces, and 1 each from Guangdong, Anhui and Jiangsu provinces, Tianjin, Beijing and Xinjiang. **Table 1** summarizes the features of the included researches.

**Table 1:** Characteristics of Included Studies.

Study	Study design	Study period	Region	Sample size	1-year survival	3-year survival	5-year survival
Guoqing Zhang 2013	Retrospective	2000-2007	Xin Jiang	1421	86.19%	46.22%	26.36%
Hong Shen 2013	Retrospective	1996-2006	Zhe Jiang	837	–	74%	68%
Yin Yuan 2015	Retrospective	1985-2011	Zhe Jiang	2454	–	–	62.6%
Bin Xu 2016	Retrospective	1993-2014	Tian Jin	812	–	–	81.9%
Jianguo Chen 2017	–	1972-2011	Jiang Su	6035	52.91%	–	27.83%
Aiping Zhou 2017	Retrospective	2005-2008	Bei Jing	627	–	–	76.37%
Xinxiang Li 2018	Retrospective	2008-2013	Shang Hai	5047	–	85.2%	77.1%
Chengyong Qin 2018	Retrospective	2000-2016	Shan Dong	4080	–	–	67.71%
Fuzhong Xue 2018	–	2010-2016	Shan Dong	2749	–	–	65.87%
Xiaopan Li 2019	–	2002-2016	Shang Hai	18592	80.73%	64.74%	52.37%
Xiaoqu Shu 2019	Retrospective	1996-2006	Shang Hai	890	–	65.9%	58.4%
Gewen Tan 2019	Retrospective	2009-2015	Shang Hai	735	–	77.21%	65.84%
Shangyi Ren 2019	Retrospective	2013-2019	Liao Ning	1315	94.19%	79.65%	65.8%
Jianghua Yang 2020	Retrospective	2012-2018	An Hui	3138	–	–	48.21%
Mingliang Zhang 2021	Retrospective	1994-2019	Guang Dong	13328	–	79.9%	71.5%
Jianmin Xu 2021	RCT	2008-2012	Shang Hai	688	–	–	79.97%

Retrospective, Retrospective cohort study; RCT, Randomized controlled trial.

### 3.2 Survival rate

**Figure 2:**
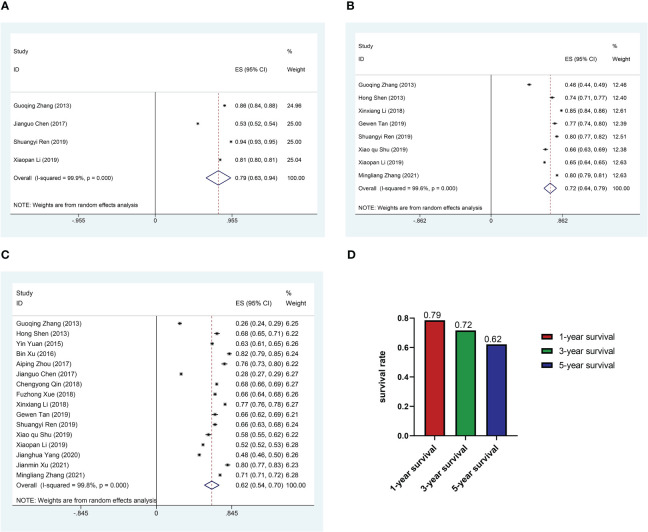
Pooled survival rate. **(A)** Forest plot of pooled 1-year survival rate in patients with colorectal cancer. **(B)** Forest plot of pooled 3-year survival rate in patients with colorectal cancer. **(C)** Forest plot of pooled 5-year survival rate in patients with colorectal cancer. **(D)** Histogram of pooled 1, 3, 5-year survival rate in patients with colorectal cancer.

The median follow-up of the 16 researches was 19.76 to 130 months. Four researches mentioned 1-year survival rate of 27363 patients; eight researches included information on 3-year survival rate of 42,165 patients; sixteen researches provided enough data to calculate 5-year survival rates. The 1-, 3- and 5-year survival rates of Chinese CRC patients summarized by the random-effect model were 0.79 (95%CI: 0.63-0.94), 0.72 (95%CI: 0.64-0.79) and 0.62 (95%CI: 0.54-0.70) respectively (**Figure 2**).

### 3.3 Subgroup analysis

**Figure 3:**
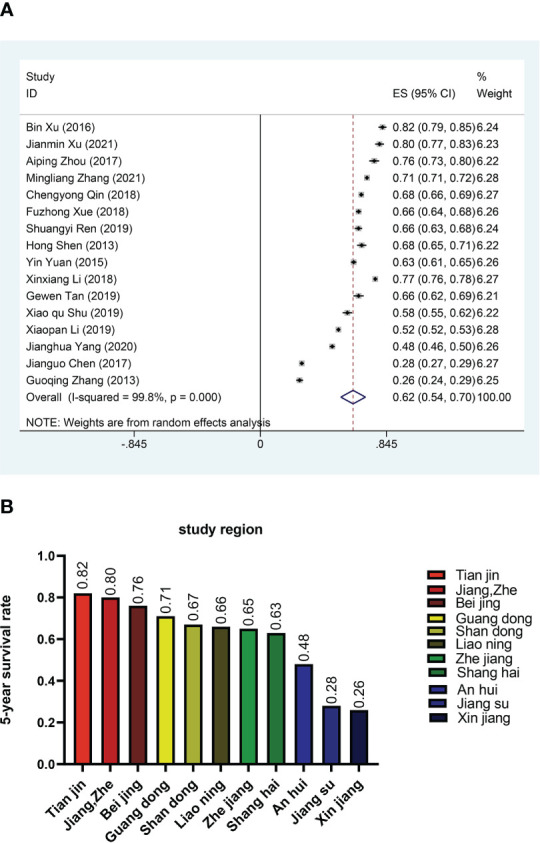
Survival rates in different regions **(A)** Forest plot of survival rates in different regions. **(B)** Histogram of survival rates in different regions.

**Figure 4:**
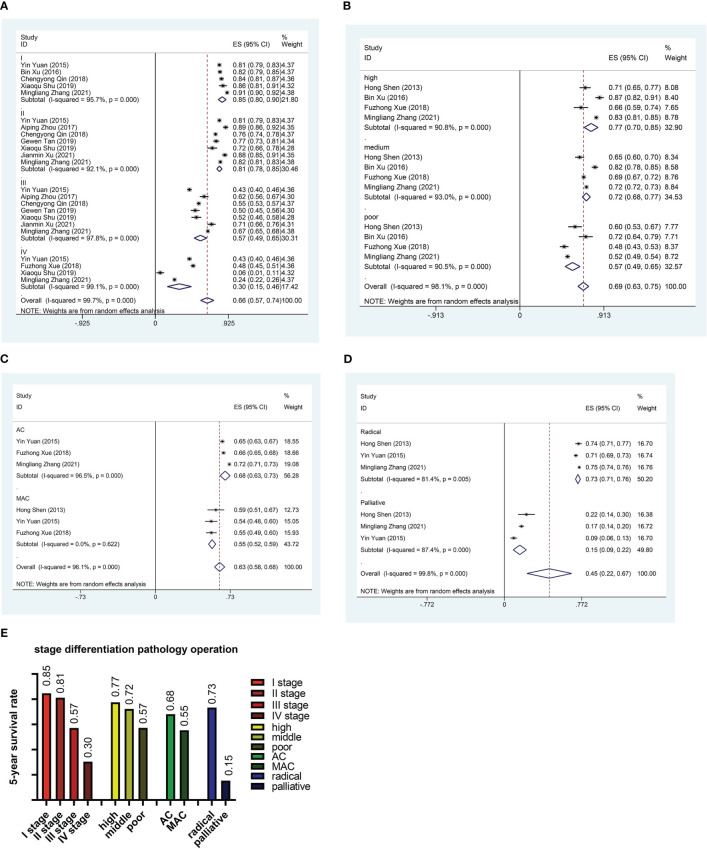
Subgroup analysis. **(A)** Forest plot of subgroup analysis based on stage. **(B)** Forest plot of subgroup analysis based on differentiation. **(C)** Forest plot of subgroup analysis based on pathological type. **(D)** Forest plot of subgroup analysis based on surgical method. **(E)** Histogram of subgroup analysis based on stage, differentiation, pathological type and surgical method. AC, Adenocarcinoma; MAC, Mucinous adenocarcinoma.

In order to reduce heterogeneity and more deeply compare the impact of patient characteristics on survival rate, we performed the subgroup analysis. The results showed significant differences in 5-year survival rates among CRC patients in different regions. Based on regional subgroup analysis, Tianjin had the highest 5-year survival rate (0.82), followed by Beijing (0.76), Guangdong (0.71), Shandong (0.67), Liaoning (0.66), Zhejiang (0.65), Shanghai (0.63), and Xinjiang had the lowest 5-year survival rate (0.26, 95%CI:0.24-0.29) (**Figure 3**). The subgroup analysis of different stages of the cancer showed that the 5-year survival rate at stage I was 0.85 (95%CI:0.80-0.90), at stage II was 0.81 (95%CI:0.78-0.85), at stage III was 0.57 (95%CI:0.49-0.65), and at stage IV was only 0.30 (95%CI:0.15-0.46) (**Figure 4A**). Four researches directly compared 5-year survival rates among patients with different degrees of differentiation, with 5-year survival rates of 0.77 (95%CI: 0.70-0.85) and 0.72 (95%CI: 0.68 -0.77) in the highly and moderately differentiated subgroups respectively, and 0.57 (95%CI: 0.49 - 0.65) in the poorly differentiated subgroup (**Figure 4B**). In addition, according to the subgroup analysis based on different pathological types of the tumor, the 5-year survival rate was 0.68 (95%CI:0.63-0.73) in the adenocarcinoma subgroup and 0.55 (95%CI: 0.52-0.59) in the mucinous adenocarcinoma subgroup (**Figure 4C**). The 5-year survival rate of radical surgery patients in our study was 0.73(95%CI:0.71-0.76), while the 5-year survival rate of palliative surgery patients was only 0.15(95%CI:0.09-0.22) (**Figure 4D**). The histogram summarizes the 5-year survival rate of patients in subgroups with different tumor stage, differentiation, pathological type, surgical approach (**Figure 4E**). There were no significant differences in subgroup analysis based on age, sex, and tumor site, as shown in **Supplementary Figure 1** and **Supplementary Figure 2**.

### 3.4 Meta-regression analysis

We performed the meta-regression analysis to explore the potential causes of heterogeneity, fitting factors of sample size, publication year, research year and study region into a univariate model. The results showed that sample size and study region were the main causes for heterogeneity, with *P* values of 0.001 and 0.000 respectively. Meta-regression analysis based on publication year and hospitalization year of the patients found no significant heterogeneity, with *P* values of 0.075 and 0.437 (**Table S1**, **Supplementary Figure 4**).

### 3.5 Publication bias and evaluation of reference quality

**Figure 5:**
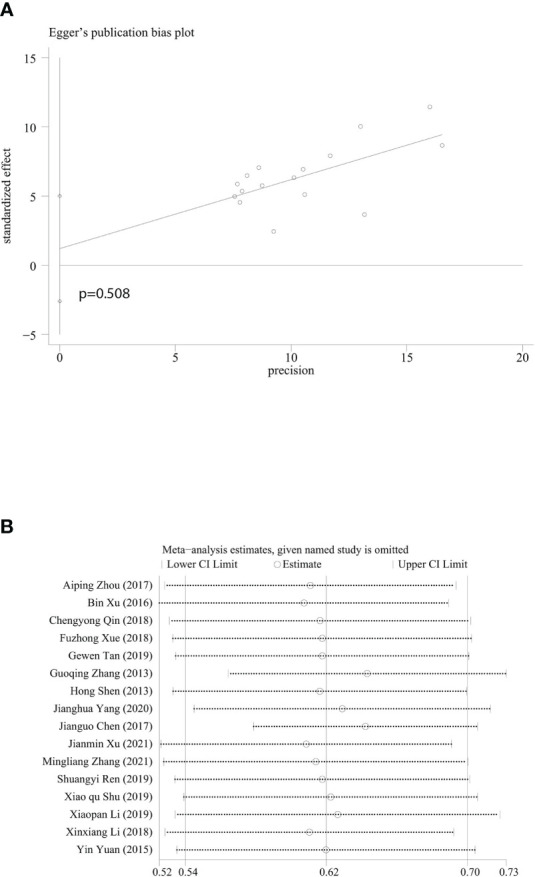
Egger’s publication bias plot and sensitivity analysis. **(A)** Egger’s publication bias plot based on 5-year survival rate. **(B)** Sensitivity analysis based on 5-year survival rate. Every horizontal line representing combined 5-year survival and the range of 95% CI after omitting study of the included studies one by one.

In the evaluation of publication bias in the included studies, we found asymmetry in funnel plot (**Supplementary Figure 4**), however, it was proven in the more sensitive Egger test that publication bias of the included researches did not exist (*P*=0.508) (**Figure 5A**). Sensitivity analysis showed that there was rather significant heterogeneity in the researches from Guoqing Zhang’s research group and Jianguo Chen’s research group (11, 15) (**Figure 5B**). We believe that heterogeneity may be due to differences in regions and publication years of the study. The economic development, medical level, lifestyle and dietary habits differs from regions, and might affect survival rates in CRC patients. As for Chen’s study, the diagnosis year for the included patients is 1993-2007, which may lead to publication bias. After experiencing regional economic development, improvement of comprehensive treatment options, and changes in healthcare and services, CRC survival rates vary from different years. However, this article can truly reflect the prognosis of CRC patients in China over the past 50 years. We then assessed the methodological quality of all included researches. In one randomized controlled trial, Cochrane Handbook for Systematic Reviews of Interventions alone was used to evaluate its methodological quality, and the risk was evaluated as low for all parts. One epidemiological research (15) provided effective survival rates for the meta-analysis, but there was not enough information for the evaluation of methodological quality, so the rate was rather low (4 stars), while the quality of rest of the researches were all equal to or higher than 5 stars (**Table S1**).

## 4 Discussion

The 1-, 3- and 5-year survival rates of patients with CRC in China were obtained in this meta-analysis. Among the included studies, the pooled 1-year and 3-year survival rates were 0.79 (95%CI: 0.63-0.94) and 0.72 (95%CI: 0.64-0.79), respectively. According to a population-based data analysis, the 1-year survival rates of patients with CRC in Australia, Canada, Norway, Denmark, and the United Kingdom were 0.849, 0.835, 0.824, 0.777, and 0.747, respectively (30). The 1-year survival rates in these regions were basically consistent with our findings. The pooled 5-year survival rate of the 16 included studies was 0.62 (95% CI: 0.54-0.70). A study published in 2019 reported 5-year survival rates for CRC patients in Australia, Canada, Denmark, Ireland, New Zealand, Norway and the United Kingdom. The highest survival rate was 0.708 (95%CI: 0.70-0.715) in Australia, and the lowest was 0.589 (95%CI: 0.586-0.593) in The UK (31). The 5-year survival rate of CRC in China was close to most European countries. Comparatively, Japan, an Asian country, has a 5-year survival rate of 0.73 for CRC, 0.628 for South Korea; 0.61 for Iran; 0.582 for Jordan and 0.342 for Malaysia (32–36). The 5-year survival rate in China was still lower than Japan and South Korea, but the gap was gradually narrowing.

Many factors can affect the prognosis of CRC patients, such as economic status, cancer stage, histological type, tumor location, and age at diagnosis (37, 38). The incidence and mortality of CRC in regions with high Human Development Index (HDI) in the world are at least twice as high as those in regions with low HDI (39). We found that region had an effect on 5-year survival. CRC is a multifactorial disease caused by lifestyle, genetic and environmental factors (40–42). China has a wide geographical area, and different regions have different lifestyles and dietary habits, which lead to different survival rates of CRC in different regions. A study from Malaysian also confirmed wide regional differences in CRC survival (43). In our research, Tianjin had the highest 5-year survival rate (0.82, 95%CI: 0.79-0.85), and Xinjiang had the lowest 5-year survival rate (0.26, 95%CI:0.24-0.29). Due to the small number of included studies, only one study mentioned the 5-year survival rate in Xinjiang (0.26), so it cannot comprehensively represent the 5-year survival rate of patients with CRC in the entire Xinjiang region. The high 5-year survival rate in Tianjin was also due to the fact that the study targeted at stage I CRC patients. For CRC, screening is conducive to early diagnosis and can improve the survival rate of CRC patients. Thus, CRC screening has been recommended in clinical practice guidelines in many countries (44, 45). The implementation of the National Danish Colorectal Cancer Screening Programme was considered a success and the programme was hopefully in the process of reducing colorectal cancer morbidity and mortality in Denmark (46). Policies vary from region to region, and some cities have cancer screening programs in place. For example, in Shanghai, as early as 2013, the CRC screening program was incorporated into community medical services, which greatly improved the 5-year survival rate of patients with colorectal cancer in Shanghai (20).

The pathological stage of tumor at the initial diagnosis is the most important factor in determining the behavior and prognosis for CRC, and mortality rises with tumor stage (47, 48). In our study, the pooled 5-year survival rate at stage I was 0.85, 0.81 at stage II, 0.57 at stage III, and 0.30 at stage IV. It is difficult for patients at stage III and IV to achieve radical cure of the disease and reduce the survival rate. Due to the deeper tumor infiltration, the cancer cells involve surrounding tissues, organs and regional lymph nodes. Rajaa Chatila’s study also confirmed that stage was a major determinant of prognosis in patients with CRC. After adjusting for age and gender in his study, there was a highly significant difference between stage IV patients and stage I patients (HR = 8.81, 95% CI: 3.20-24.22, *p* = 0.000) (49). Incorporating the surgical method (radical or palliative) into the nomogram model can visually display that the surgical method was an independent prognostic factor affecting the overall survival rate of CRC patients (50). Therefore, the 5-year survival rate of radical surgery patients in our study was 0.73, while the 5-year survival rate of palliative surgery patients was only 0.15. In the subgroup analysis, the 5-year survival rate of the well-differentiated subgroup was 0.77, the 5-year survival rate of the moderately differentiated subgroup was 0.72, and the 5-year survival rate of the poorly differentiated subgroup was 0.57. The existence of prognostic differences between mucinous and non-mucinous colorectal carcinoma, mucinous differentiation results in increased hazard of death (51). In our results, the 5-year survival rates for adenocarcinoma and mucinous adenocarcinoma were 0.68 and 0.55, respectively. Mucinous adenocarcinoma showed a lower 5-year survival rate. The reason may be that mucinous adenocarcinoma, a pathological type, has different characteristics from adenocarcinoma, including younger patients, an advanced stage at diagnosis, and more prone to metastasis (52).

The results of subgroup analysis showed that there was no significant difference in the 5-year survival rate with different age, gender, and tumor site. The 5-year survival rates of patients <60 years and ≥60 years were 0.70 and 0.67, while 0.67 and 0.70 in male and female, respectively. Primary tumor site affects prognosis in patients with CRC. Although there have been studies reporting that right-sided colon cancer has worse overall survival compared to left-sided colon cancer, in our study, there was no significant difference between the two (0.74 *vs*. 0.71) (53–57). The 5-year survival rates of colon and rectal cancer subgroups were almost equal (0.70 *vs*. 0.69). It may be related to the fact that we included too few studies, with only 6 studies summarizing 5-year survival in patients with colon/rectal cancer and only 3 studies comparing 5-year survival in the left colon versus the right colon.

One of the limitations of this study is that although we included up to 62748 patients, the number of studies included was small. In order to minimize publication bias and make the included researches more representative, we chose to include researches from large study centers. We determined a threshold of 500 cases based on the actual number of patients in the articles. We hope that this threshold can reduce bias. Many studies did not mention 1-year and 3-year survival, resulting in only 4 studies summarizing 1-year survival and 8 studies summarizing 3-year survival. The information about the survival rate in many studies was not comprehensive. We obtained the survival rate by calculation, so there may be minor deviations. Lynch syndrome is a common CRC-related genetic syndrome. Unfortunately, available research data could not support comparisons of survival rates for genetic and non-hereditary colorectal cancers. The vast majority of Chinese cities have not published studies on CRC survival rates, so it was impossible to summarize the survival rates in various regions of China. Moreover, there were too few related studies in some areas, which is prone to the phenomenon of generalization like the 5-year survival rate in Xinjiang. Summarized information on survival rates of CRC patients in China is lacking. Our study complements the 5-year survival rate information for CRC in different regions and different clinicopathological features in China.

## 5 Conclusions

The 5-year survival rate in China is close to that of most European countries, but still lower than Japan and South Korea, and the gap is gradually narrowing. Region, stage, differentiation, pathological type, and surgical approach can affect 5-year survival in colorectal cancer.

## Data availability statement

The original contributions presented in the study are included in the article/**Supplementary Material**. Further inquiries can be directed to the corresponding author.

## Author contributions

HL and JL contributed to the research concept and design. RW and XW retrieved and filtered articles, and XP and BX extracted data. RW and JL analyzed the data. RW, XW, JS, and ST explained the data. RW and JL drafted manuscript. HL and JL contributed to critical revision of the manuscript. All authors contributed to the article and approved the submitted version.
