# Supplementary material for: Survival rate of colorectal cancer in China: A systematic review and meta-analysis
Source: Front Oncol. 2023 Mar 3;13:1033154. doi: 10.3389/fonc.2023.1033154 (PMC10020492; doi:10.3389/fonc.2023.1033154)
Supplement: Supplementary file 1 [file DataSheet_1.docx]

Supplementary Material

# Supplementary Figures and Tables

## Supplementary Figures


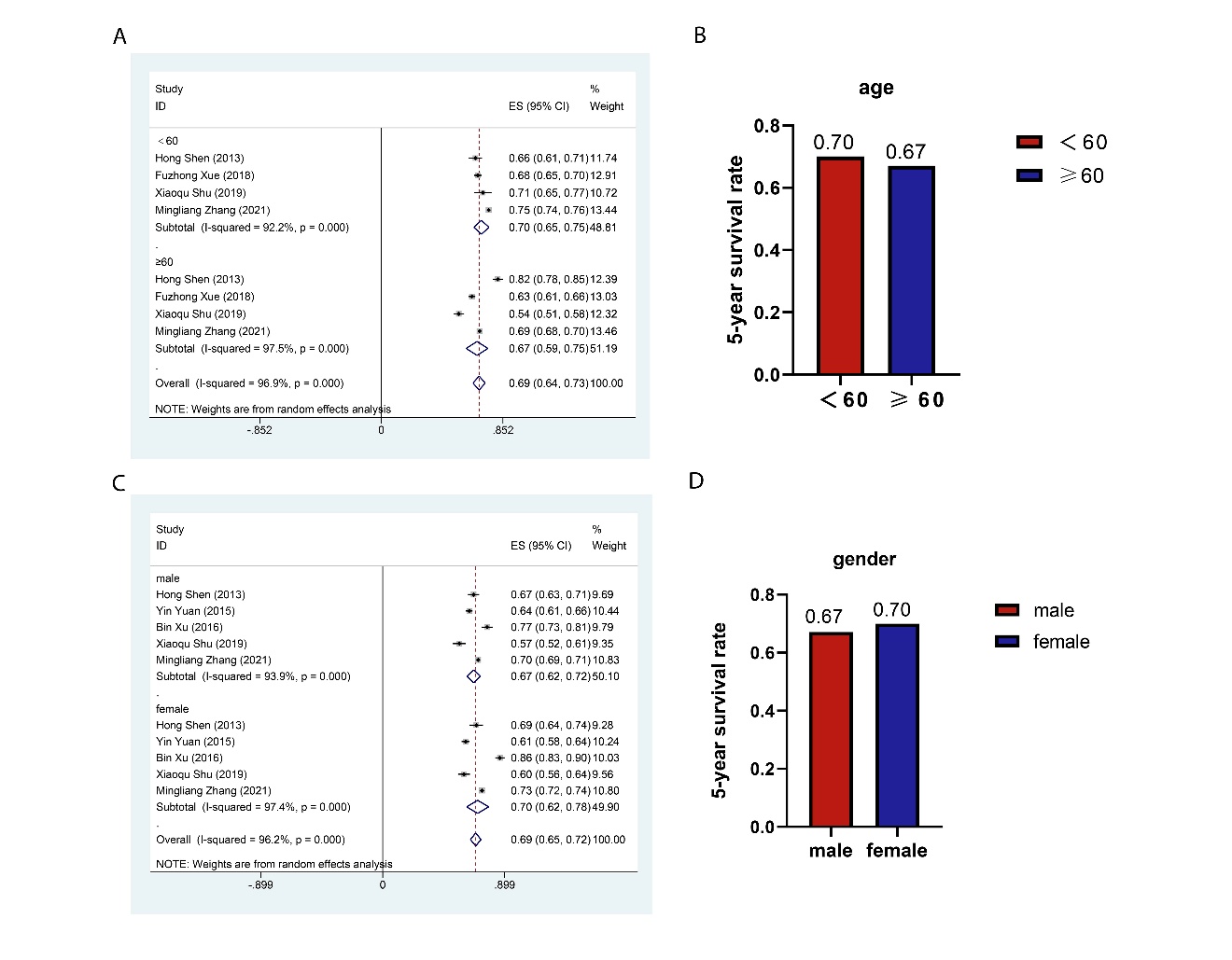


**Supplementary Figure 1.** Subgroup analysis. **(A),** Forest plot of subgroup analysis based on age. **(B),** Histogram of subgroup analysis based on age. **(C),** Forest plot of subgroup analysis based on gender. **(D),** Histogram of subgroup analysis based on gender.


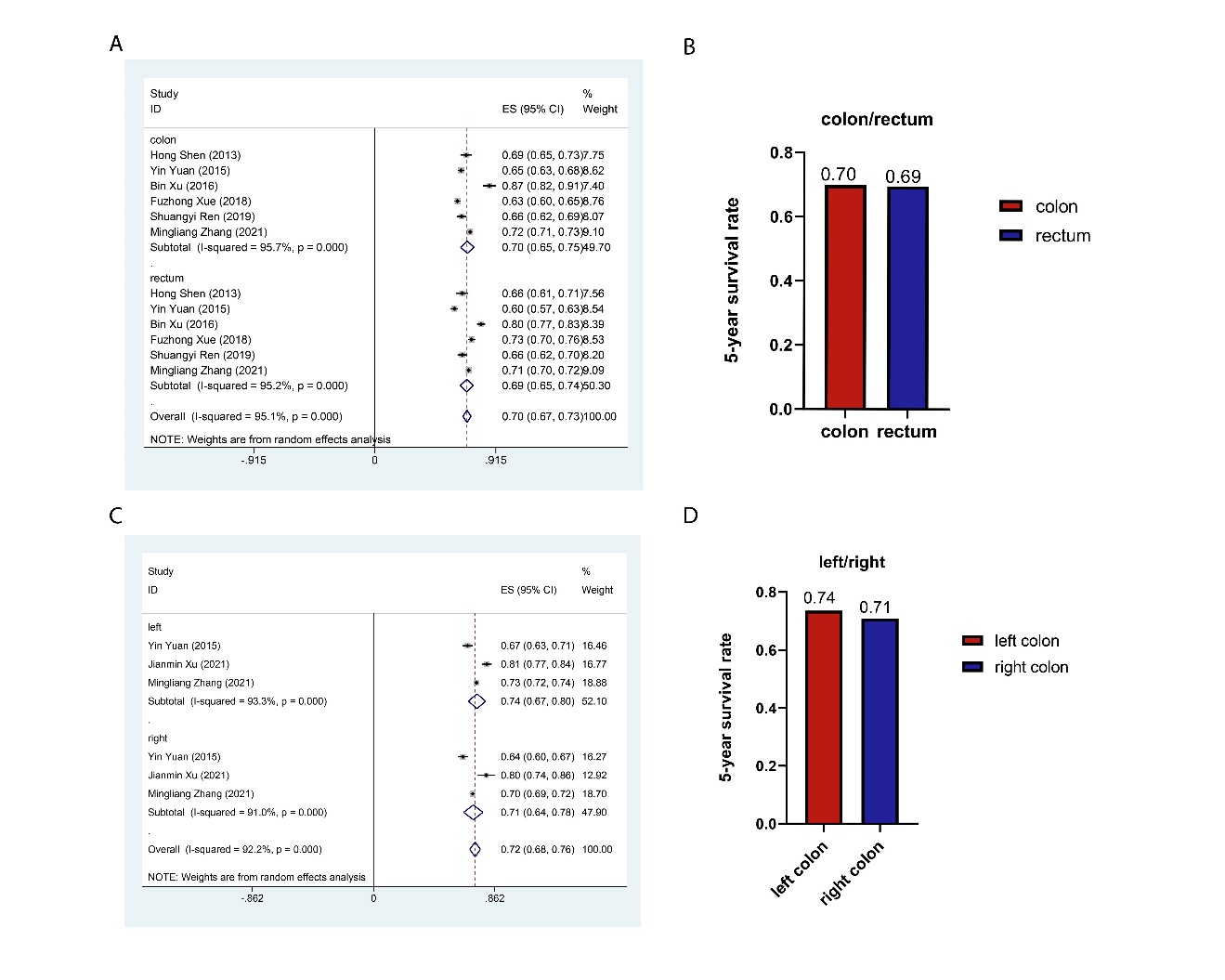


**Supplementary Figure 2.** Subgroup analysis. **(A),** Forest plot of subgroup analysis based on colon cancer or rectum cancer. **(B),** Histogram of subgroup analysis based on colon cancer or rectum cancer. **(C),** Forest plot of subgroup analysis for tumor location is left colon or right colon. **(D),** Histogram of subgroup analysis for tumor location is left colon or right colon.


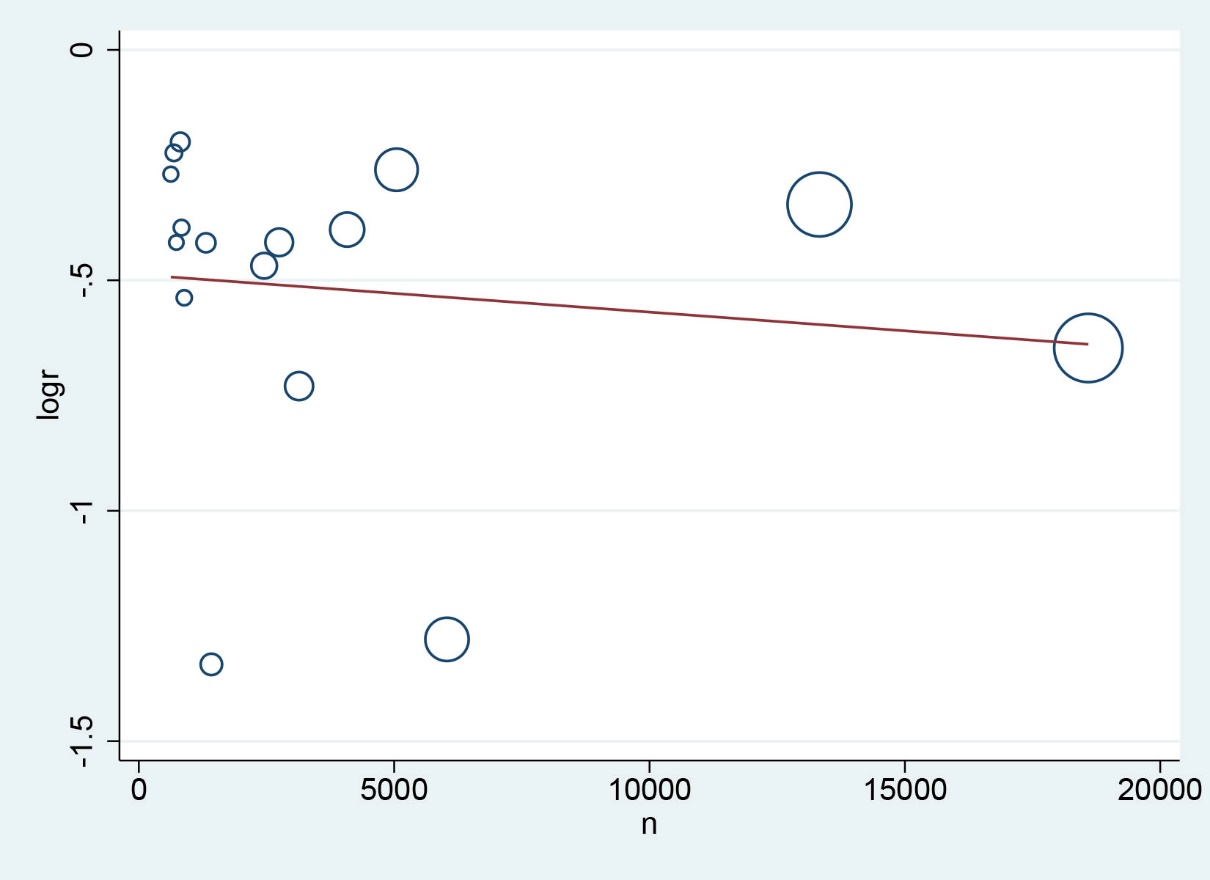


**Supplementary Figure 3.** Result of Meta-regression for 5-years Survival Rate Based on Sample Size.


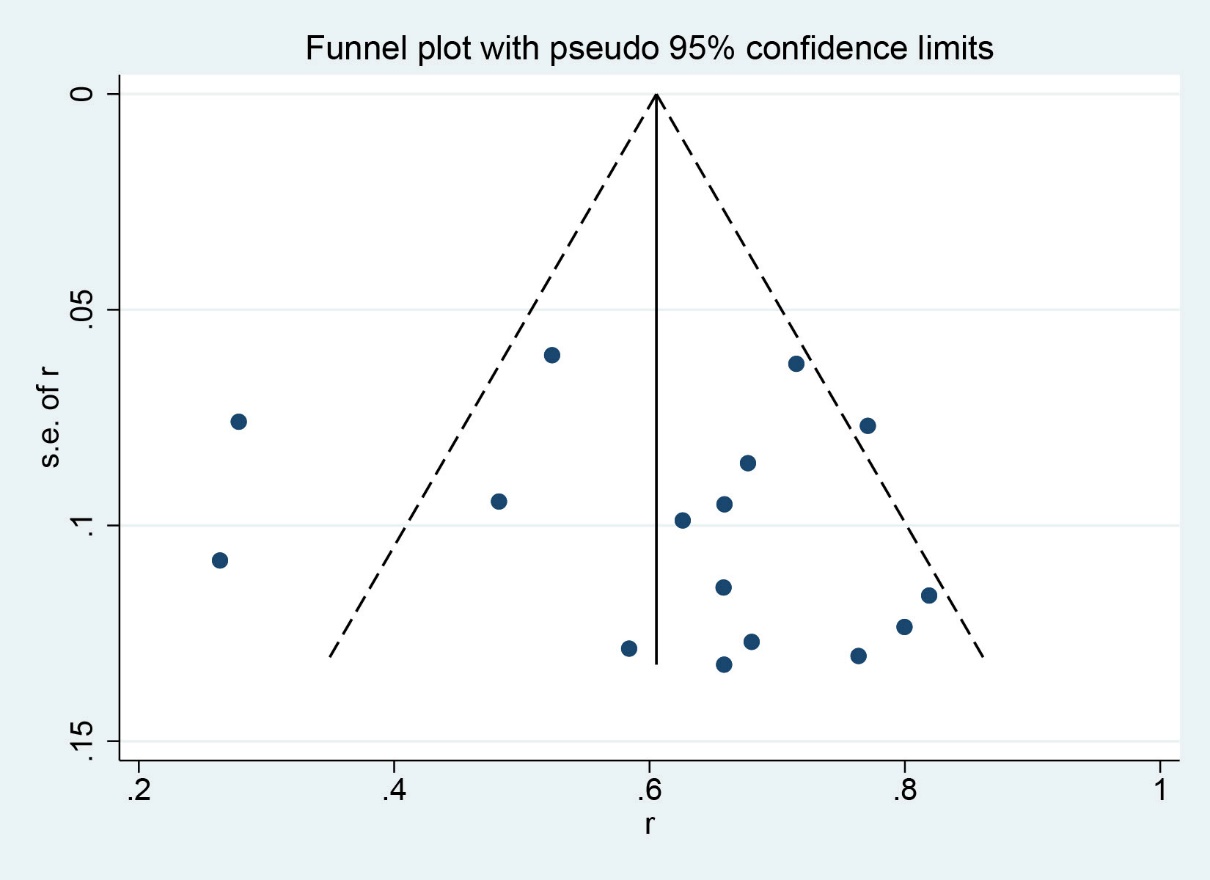


**Supplementary Figure 4.** Funnel plot based on 5-year survival rate

## Supplementary Tables

| Inclusion study | NOS  Selection | NOS  Comparability | NOS  Outcome | Total  (Max 9) |
| --- | --- | --- | --- | --- |
| Mingliang Zhang 2021 | ⭐⭐ | ⭐ | ⭐⭐ | 5 |
| Jianghua Yang 2020 | ⭐⭐ | ⭐ | ⭐⭐ | 5 |
| Shuangyi Ren 2019 | ⭐⭐ | ⭐⭐ | ⭐ | 6 |
| Xiaoqu Shu 2019 | ⭐⭐ | ⭐ | ⭐⭐ | 5 |
| Gewen Tan 2019 | ⭐⭐ | ⭐ | ⭐⭐ | 5 |
| Xiaopan Li 2019 | ⭐⭐⭐⭐ | ⭐ | ⭐⭐ | 7 |
| Xinxiang Li 2018 | ⭐⭐ | ⭐⭐ | ⭐⭐ | 6 |
| Chengyong Qin 2018 | ⭐⭐ | ⭐ | ⭐⭐⭐ | 6 |
| Fuzhong Xue 2018 | ⭐⭐ | ⭐ | ⭐⭐⭐ | 6 |
| Jianguo Chen 2017 | ⭐ | ⭐ | ⭐⭐ | 4 |
| Aiping Zhou 2017 | ⭐⭐ | ⭐ | ⭐⭐ | 5 |
| Bin Xu 2016 | ⭐⭐ | ⭐ | ⭐⭐⭐ | 6 |
| Yin Yuan 2015 | ⭐⭐ | ⭐ | ⭐⭐ | 5 |
| Guoqing Zhang 2013 | ⭐⭐ | ⭐ | ⭐⭐⭐ | 6 |
| Hong Shen 2013 | ⭐⭐ | ⭐ | ⭐⭐⭐ | 6 |

**Supplementary Table 1**. Assessment of Study Quality Using the Newcastle-Ottawa Scale for Cohort Studies. The Newcastle-Ottawa scale (NOS) which classifies individual studies as having low or high risk of bias across 3 domains: selection, comparability, and outcome.


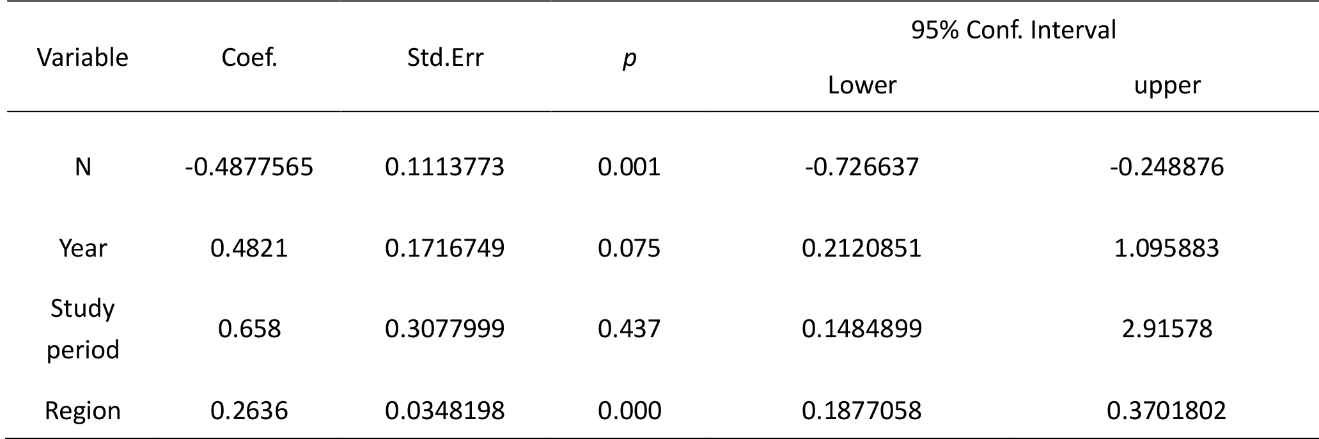


**Supplementary Table 2.** Result of Meta-regression for 5-year Survival Rate Based on Sample Size, Year of Publication, Study Period and Region.
